# Effect of remdesivir post-exposure prophylaxis and treatment on pathogenesis of measles in rhesus macaques

**DOI:** 10.1038/s41598-023-33572-7

**Published:** 2023-04-20

**Authors:** Nadine A. Peart Akindele, Laharika Dasharath Katamoni, Jacqueline Brockhurst, Shristi Ghimire, San Suwanmanee, Lisa Pieterse, Kelly A. Metcalf Pate, Elaine Bunyan, Roy Bannister, Tomas Cihlar, Danielle P. Porter, Diane E. Griffin

**Affiliations:** 1 Division of Pediatric Infectious Diseases, Johns Hopkins University School of Medicine, Baltimore, MD 21218 USA; 2 W. Harry Feinstone Department of Molecular Microbiology and Immunology, Johns Hopkins Bloomberg School of Public Health, 615 N. Wolfe St, Rm E5636, Baltimore, MD 21205 USA; 3 Zanvyl Krieger School of Arts and Sciences, Johns Hopkins University, Baltimore, MD 21205 USA; 4 Department of Molecular and Comparative Pathology, Johns Hopkins University School of Medicine, Baltimore, MD 21218 USA; 5 Gilead Sciences Inc., Foster City, CA 94404 USA; 6 United States Food and Drug Administration, Silver Spring, MD 20993 USA; 7 BioCheck, Inc., South San Francisco, CA 94080 USA; 8 Department of Epidemiology, Faculty of Public Health, Mahidol University, Bangkok, Thailand; 9 Division of Comparative Medicine, Massachusetts Institute of Technology, Cambridge, MA 02139 USA

**Keywords:** Measles virus, Viral pathogenesis, Antimicrobials, Pathogens, Virology

## Abstract

Measles is a systemic disease initiated in the respiratory tract with widespread measles virus (MeV) infection of lymphoid tissue. Mortality can be substantial, but no licensed antiviral therapy is available. We evaluated both post-exposure prophylaxis and treatment with remdesivir, a broad-spectrum antiviral, using a well-characterized rhesus macaque model of measles. Animals were treated with intravenous remdesivir for 12 days beginning either 3 days after intratracheal infection (post-exposure prophylaxis, PEP) or 11 days after infection at the onset of disease (late treatment, LT). As PEP, remdesivir lowered levels of viral RNA in peripheral blood mononuclear cells, but RNA rebounded at the end of the treatment period and infectious virus was continuously recoverable. MeV RNA was cleared more rapidly from lymphoid tissue, was variably detected in the respiratory tract, and not detected in urine. PEP did not improve clinical disease nor lymphopenia and reduced the antibody response to infection. In contrast, LT had little effect on levels of viral RNA or the antibody response but also did not decrease clinical disease. Therefore, remdesivir transiently suppressed expression of viral RNA and limited dissemination when provided as PEP, but virus was not cleared and resumed replication without improvement in the clinical disease parameters evaluated.

## Introduction

Measles is a highly contagious childhood exanthem. It is caused by measles virus (MeV), an enveloped, non-segmented, negative sense RNA virus that belongs to the *Paramyxoviridae* family. Infection with MeV yields durable protective immunity that is postulated to be generated through the persistence of viral RNA in lymphoid tissue for months after infection^1,2^. The live attenuated MeV vaccine developed in the mid-twentieth century by in vitro passage of wild-type (WT) MeV^3^ has prevented an estimated 23.2 million deaths globally^4^. Despite declaration of measles elimination in the US in 2000, nation-wide outbreaks occurred in 2019 and global measles cases increased 167% between 2016 and 2018. In 2018, there were 353,236 cases and an estimated 142,300 deaths reported worldwide^4^, primarily in children less than 5 years of age^5^. Lapses in MeV vaccination due to disruption of routine immunization programs during the severe acute respiratory coronavirus 2 (SARS-CoV-2) pandemic have exacerbated this problem resulting in current estimates of 250,000 unvaccinated kindergarteners in the US^6,7^.

Currently, the management of measles is primarily with supportive measures, vitamin A supplementation, and antibiotic treatment of secondary bacterial infections^8^. The broad-spectrum antiviral, GS-5734, also known as remdesivir, has in vitro activity against several pathogenic plus-strand and minus-strand RNA viruses such as coronaviruses (including SARS-CoV-2), paramyxoviruses (including MeV), and filoviruses^9–11^. Remdesivir has in vivo efficacy in non-human primates (NHPs) for treatment of lethal Nipah^12^, Ebola^13^ and Marburg^14^ virus infections, as well as non-lethal SARS-CoV-2^15^ and Middle East respiratory syndrome (MERS-CoV)^16^ virus infections. Remdesivir has been used in clinical trials against Ebola virus disease^17^ and is approved and widely used for the treatment of COVID-19 caused by SARS-CoV-2^18–21^. To determine whether remdesivir might be useful for the treatment of measles, we evaluated the effect of drug administration on development of clinical disease, both early (post-exposure prophylaxis, PEP) and at the time of rash onset (late treatment, LT), in rhesus macaques infected with WT MeV. We demonstrate that initiation of intravenous treatment with remdesivir 3 days after WT MeV infection for a total of 12 days suppressed levels of viral RNA during treatment. However, virus remained recoverable by cocultivation of peripheral blood mononuclear cells (PBMCs) and viral RNA levels rebounded upon cessation of treatment, eventually clearing with a time course similar to viral RNA detected in untreated controls. Clinical disease was not improved compared to untreated control animals that received infusions of vehicle; however, clearance of viral RNA from lymphoid tissue was more rapid and production and maturation of antiviral antibody responses were impaired. Initiation of treatment at 11 days after infection did not affect levels of viral RNA during the time of treatment or development of antiviral antibodies.

## Results

### Remdesivir treatment did not alter clinical disease

**Figure 1:**
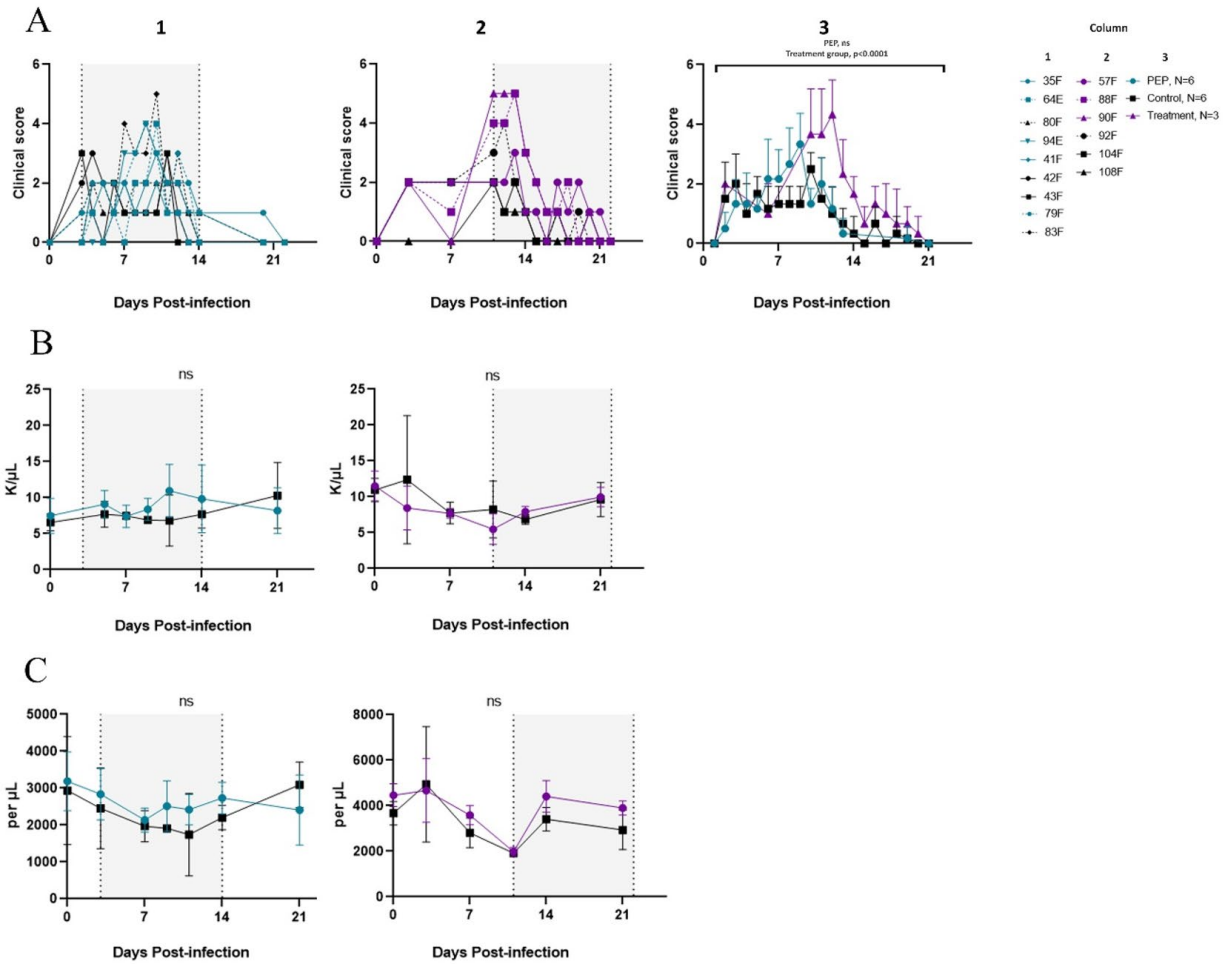
Clinical scores and laboratory results for MeV-infected macaques treated with remdesivir during the acute phase of disease. (**A**) Clinical scores obtained using a 10-parameter scale by two study veterinarians, blinded to treatment group. Column 1: Individual data from PEP study group and untreated controls; Column 2: Individual data from late treatment study group and untreated controls; Column 3: Averaged data with pooled untreated control group. (**B**) White blood cell count and (**C**) Absolute lymphocyte count (ALC) from DPI 0 to DPI 21. Note: number of untreated control monkeys for group-specific data is N = 3 each. Shaded areas indicate treatment periods.

Treatment with intravenous remdesivir was initiated either 3 days (PEP; N = 6) or 11 days (LT; N = 3) after intratracheal infection with WT MeV and continued for 12 days. Untreated control animals received similarly administered diluent (N = 3 for each group). Macaques were monitored by two study veterinarians blinded to treatment group and scored for energy level, mentation, appetite, rash, fever, conjunctivitis, Koplik spots, lymphadenopathy, urine output, and stool changes using a 10-parameter scale yielding a score between 0 and 13 (Table S1). Noted clinical features included rash, Koplik spots (Fig. S1) and lymphadenopathy most often present on day post-infection (DPI) 11 and 12. The highest documented temperature was 102.7°F by rectal thermometer in an untreated macaque (108F). In the PEP study group, the mean clinical score was higher than that of the untreated macaques throughout most of the study, but this difference was not significant (Fig. 1A). Clinical scores of the LT study group macaques were higher than those of the untreated control group macaques (Fig. 1A, *p* < 0.0001) due to the presence of more severe rashes and lymphadenopathy in two out of three macaques (88F and 90F) in the LT group. Macaques in all groups recovered and scores returned to baseline by DPI 22 after which clinical scoring was discontinued.

### Remdesivir treatment did not prevent lymphopenia

Complete blood counts with differentials and routine laboratory chemistries were monitored for MeV-associated lymphopenia other changes potentially associated with infection or drug toxicity (Fig. 1B,C; Fig. S2). In both the PEP and control groups, total WBCs initially rose (DPI 5 PEP mean 9.1 K/µl ± 1.89 and untreated control mean 7.7 K/µl ± 1.8, p > 0.05, Figs. 1B, S2A). While the untreated control group WBCs subsequently decreased to a nadir of 6.82 K/µl ± 3.54 on DPI 11, the PEP group WBC increased to a peak of 10.95 K/µl ± 3.68, although this difference was not significant. Absolute lymphocyte count (ALC) decreased to a mean nadir of 2136/µl ± 324 on DPI 7 in the PEP study group and 1740/µl ± 1116/µl in the untreated control group before increasing back to baseline (*p* > 0.05) (Figs. 1C, S2B). There were no differences in mean absolute neutrophil counts, hemoglobin concentrations, platelet counts (nadir on DPI 11 of 90 K/µl in PEP group and 83 K/µl in untreated control group), alanine aminotransferase or aspartate amino transferase during the treatment period (Fig. S2C-G).

For the LT and untreated control groups, total WBCs were similar throughout the monitored time period (Figs. 1B, S2A). ALC reached a nadir on DPI 11 in both groups (mean treatment ALC 1985/µl ± 191/µl and untreated control 1917/µl ± 121/µl, Fig. 1C) then gradually increased, though to higher levels in the LT group compared to the untreated control group (*p* = 0.0261) (Fig. S2B). There were no differences in mean absolute neutrophil counts, hemoglobin, platelet counts, alanine aminotransferase or aspartate amino transferase during or after treatment (Fig. S2C-G).

### Early administration of remdesivir decreased levels of viral RNA during treatment, but infectious virus was still recoverable

**Figure 2:**
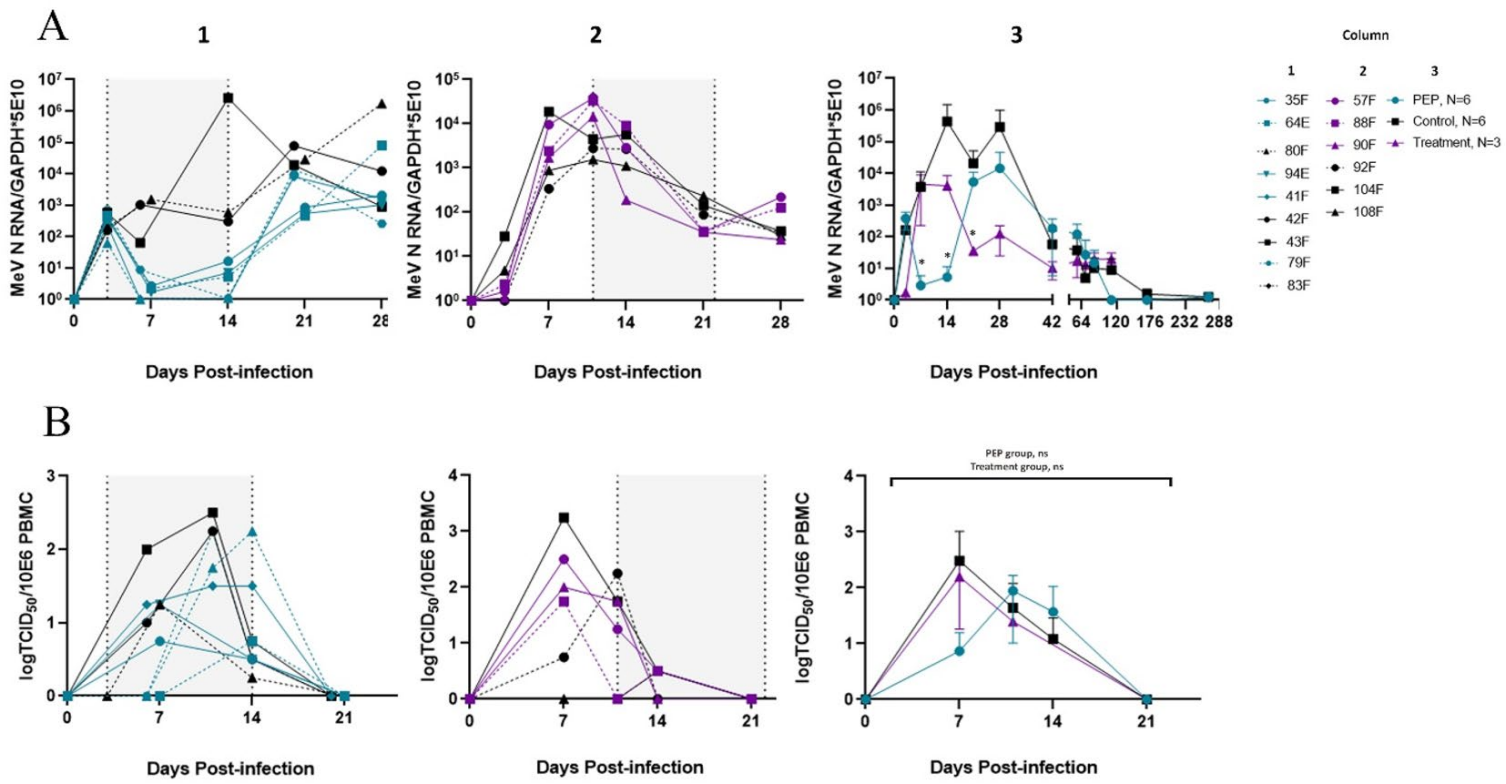
MeV N RNA and infectious virus for MeV-infected macaques treated with remdesivir. (**A**) Measles virus N gene ribonucleic acid (RNA) kinetics and (**B**) Infectious virus measured by TCID_50_ after co-cultivation of PBMCs isolated from infected macaques during the viremia. Column 1: Individual data from PEP study group and untreated controls during acute infection and treatment; Column 2: Individual data from late treatment study group and untreated controls during acute infection and treatment. Shaded areas indicate treatment periods. Column 3: Averaged data with pooled untreated control group for entire follow-up periods. Asterisk indicates p < 0.05 for the time point.

Viral RNA and virus capable of replication in vitro were measured to monitor presence of MeV. PEP with remdesivir initially decreased levels of viral RNA in PBMCs compared to untreated controls in all animals and two macaques, 79F and 41F, had no detectable MeV RNA 7 days after infection. Mean levels of viral RNA were significantly lower in the PEP treatment group as compared to untreated controls at times of peak viremia, DPI 7 and 14 (*p* = 0.0022) (Fig. 2A). However, after drug administration ended (DPI 14) amounts of viral RNA rebounded to approximately the same levels as were present in untreated macaques and then was gradually cleared over the next 2–4 months. When PBMCs obtained during drug administration were cocultured with Vero/hSLAM cells in the absence of remdesivir, syncytia developed, and infectious virus was recovered at levels comparable to those of untreated control macaques (Fig. 2B). Peak viremia in untreated macaques occurred around DPI 11 and infectious virus was cleared by DPI 21 as previously observed^22^ (Fig. 2B). In macaques that received PEP with remdesivir, the timing of peak viremia was more variable, but overall amounts of virus recovered were not different from those in untreated animals, and infectious virus was no longer recovered from any animal by DPI 21 (Fig. 2B).

**Figure 3:**
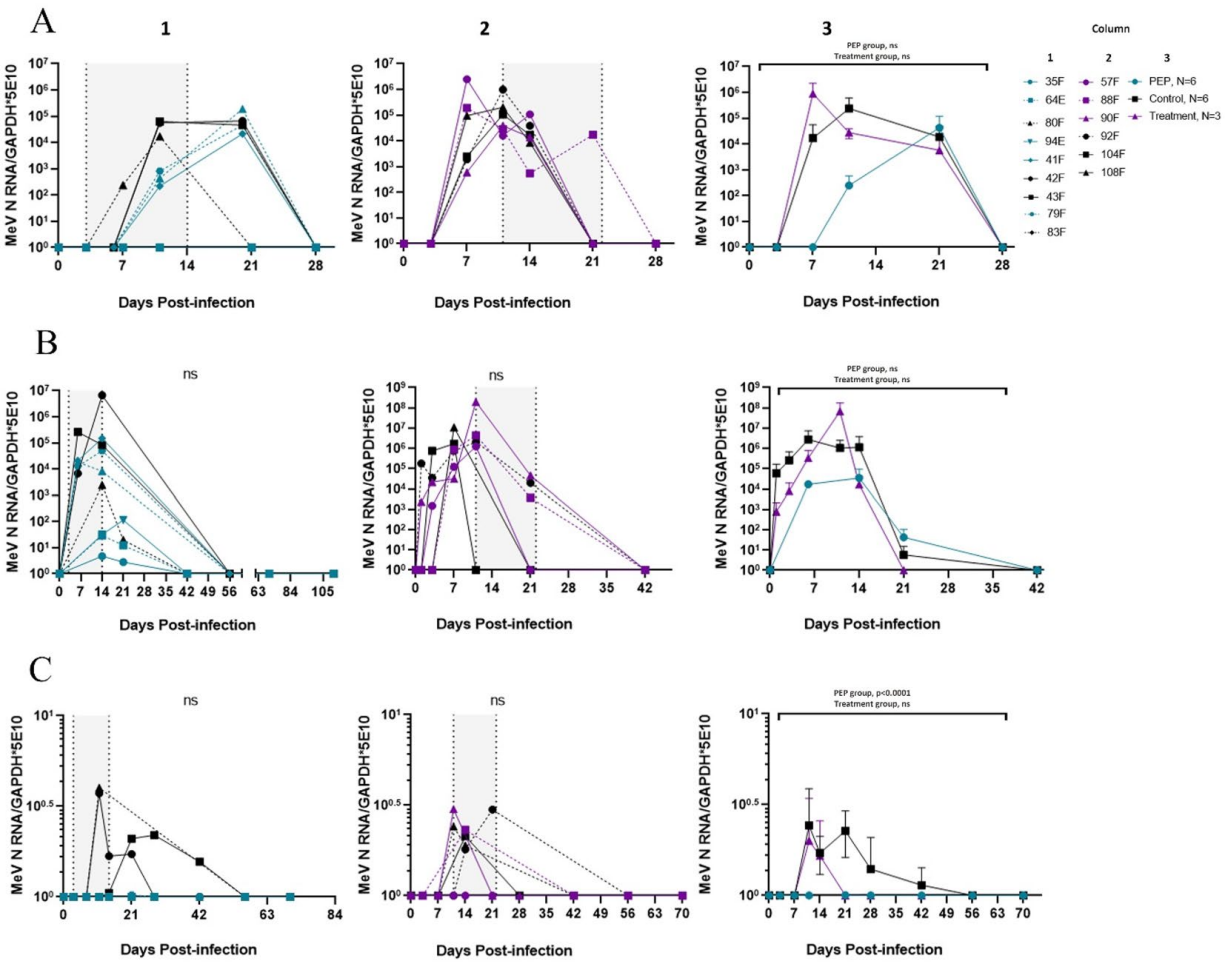
MeV N RNA in respiratory samples and urine. MeV N RNA detected by RT-qPCR in RNA extracted from (**A**) Nasal swab pellet cells, (**B**) 1 × 10^6^ BAL cells and (**C**) urine. Column 1: Individual data from PEP study group and untreated controls; Column 2: Individual data from late treatment study group and untreated controls; Shaded areas indicate treatment periods. Column 3: Averaged data with pooled untreated controls.

The effect of remdesivir prophylaxis on levels of viral RNA in cells from nasopharyngeal swab pellets and bronchoalveolar lavage (BAL) samples was less consistent, with 3 PEP study group macaques having levels similar to untreated controls and 3 showing suppression of viral RNA expression in the respiratory tract (Fig. 3A,B). No viral RNA was detected in urine samples from PEP study group animals but was detected for about 6 weeks after infection in the urine of untreated animals (Fig. 3C, *p* < 0.0001).

Macaques in the LT study group who received treatment beginning on DPI 11, when immune-mediated virus clearance is being initiated, had significantly lower levels of viral RNA as compared to untreated controls on DPI 21 (*p* = 0.0238, Fig. 2A), but at other time points and after treatment ended, levels were similar. Infectious virus recovered was maximal on DPI 7 prior to initiation of treatment and was undetectable on DPI 21 (Fig. 2B). Levels of viral RNA in respiratory samples from LT study group macaques were similar to untreated controls but RNA was not detected after DPI 14 in urine.

**Figure 4:**
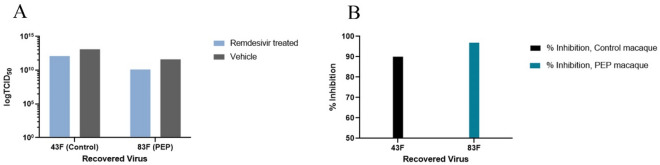
In vitro testing of remdesivir efficacy against virus recovered from PBMCs of MeV-infected macaques. (**A**) Titer of virus recovered from the PBMC of a monkey that had been treated with remdesivir (83F) compared to a monkey that was in the untreated control group (43F) 4 days after exposure in vitro to 1 µM remdesivir (blue) and vehicle (gray). (**B**) Calculated percent inhibition of recovered virus replication by remdesivir.

To confirm the susceptibility of the Bilthoven WT strain of MeV to remdesivir and determine whether virus had developed resistance in vivo, challenge virus and virus recovered from PBMCs from untreated control and PEP study group macaques were tested in vitro for susceptibility to the antiviral effects of remdesivir. The IC_50_ of remdesivir for Bilthoven was 0.66 µM (in 0.5 × 10^6^ cells after 4 days of treatment, Fig. S3) and virus recovered from macaques treated with remdesivir demonstrated viral suppression of 96% similar to that of virus recovered from untreated macaques (90%) (Fig. 4A,B).

### Remdesivir prophylaxis and late treatment shortened MeV RNA persistence in lymphoid tissue, but not bone marrow

**Figure 5:**
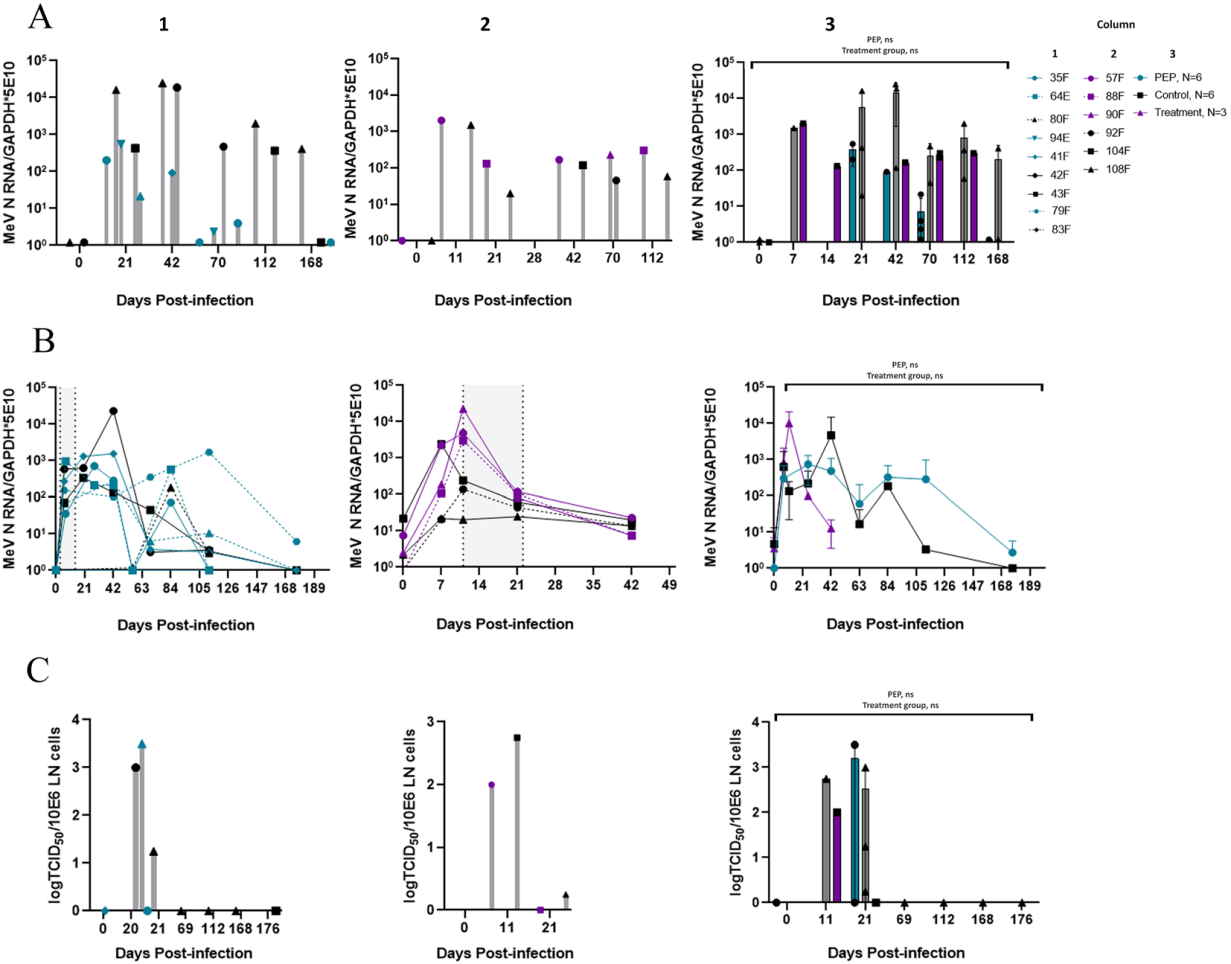
MeV N RNA in lymphoid tissue and bone marrow. MeV N RNA detected by RT-qPCR in RNA extracted from (**A**) 2 × 10^6^ LN cells and (**B**) 2 × 10^6^ BM cells. (**C**) Infectious virus measured by TCID_50_ after co-cultivation of LN cells isolated from infected macaques. Column 1: Individual data from PEP study group and untreated controls; Column 2: Individual data from late treatment study group and untreated controls. Shaded area indicates treatment period. Column 3: Averaged data with pooled untreated control group.

Because persistence of viral RNA in lymphoid tissue and loss of antibody produced by long-lived plasma cells is characteristic of untreated WT MeV infection^2,23^, macaques were followed with sampling of lymph nodes (LNs) (Fig. 5A,C) and bone marrow (BM) (Fig. 5B) for 6–8 months after infection to monitor the effect of remdesivir on clearance of MeV RNA (Fig. 5A,B) and infectious virus (Fig. 5C). At early time points (i.e., DPI 11 and 21), both viral RNA and infectious virus were present in LN cells of PEP, LT, and untreated control animals, but at later times points, only RNA was detected (Fig. 5A,C). Viral RNA in LNs decreased more rapidly in PEP study group macaques compared to LT and untreated macaques. In both PEP and LT group macaques, BM cells had similar levels of viral RNA to untreated macaques (Fig. 5B).

### Early remdesivir administration suppressed development and maturation of the antiviral antibody response

**Figure 6:**
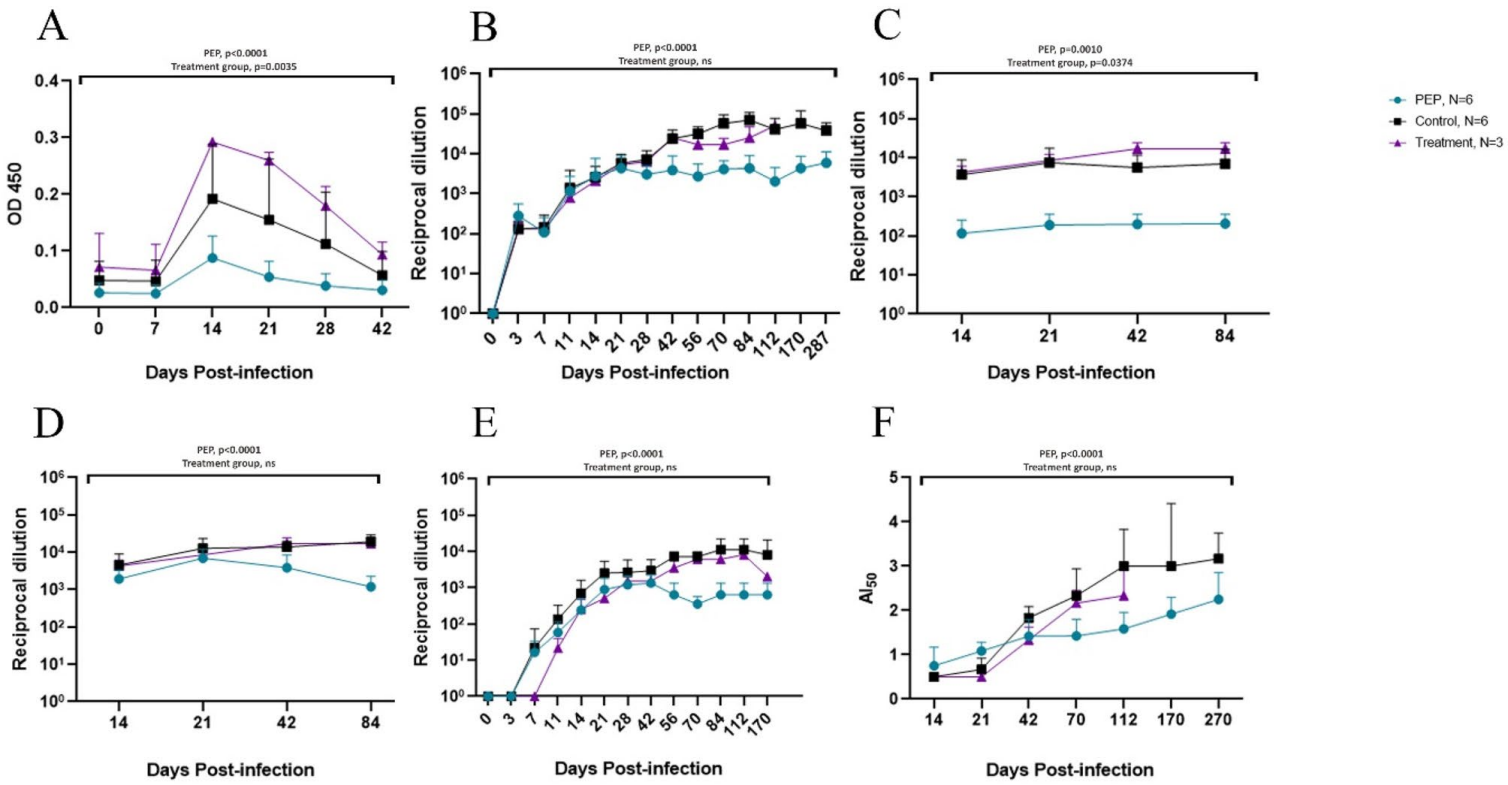
MeV-specific humoral immune response in MeV-infected macaques treated with remdesivir as compared to untreated controls. (**A**) IgM; (**B**) IgG; (**C**) Antibody to the H glycoprotein; (**D**) Antibody to the N protein; (**E**) Plaque reduction neutralization titer; (**F**) Avidity index.

To determine the effect of remdesivir on the humoral immune response to infection we evaluated the plasma levels of MeV-specific IgM and IgG binding and neutralizing antibodies (Fig. 6). Initiation of responses was similar with maximal levels of IgM at DPI 14, though at significantly lower levels in PEP group macaques (Fig. 6A, *p* < 0.0001). IgG levels rapidly rose through DPI 21, however, after DPI 28 in PEP-treated macaques mean IgG titer plateaued at levels lower than titers achieved by untreated animals (*p* < 0.001, Fig. 6B). This represented lower levels of binding IgG antibody to both the hemagglutinin (H; *p* = 0.001) and nucleocapsid (N; *p* < 0.0001) MeV proteins (Fig. 6C,D). Most MeV-specific neutralizing antibody is directed towards the H glycoprotein^24^ and in PEP-treated macaques, neutralizing antibody also plateaued at levels lower than untreated animals (Fig. 6E; *p* < 0.0001). Avidity maturation was also less robust with a lower mean AI_50_ (2.3 in the PEP study group vs. 3.2 in untreated animals at 9 months after infection; Fig. 6F, *p* < 0.0001). In general, MeV-specific antibody production and maturation was not adversely affected by treatment initiated at the onset of disease in the LT group (Fig. 6).

### Late treatment with remdesivir did not alter induction of MeV-specific lymphocytes producing interferon-γ (IFN-γ) or IL-17

**Figure 7:**
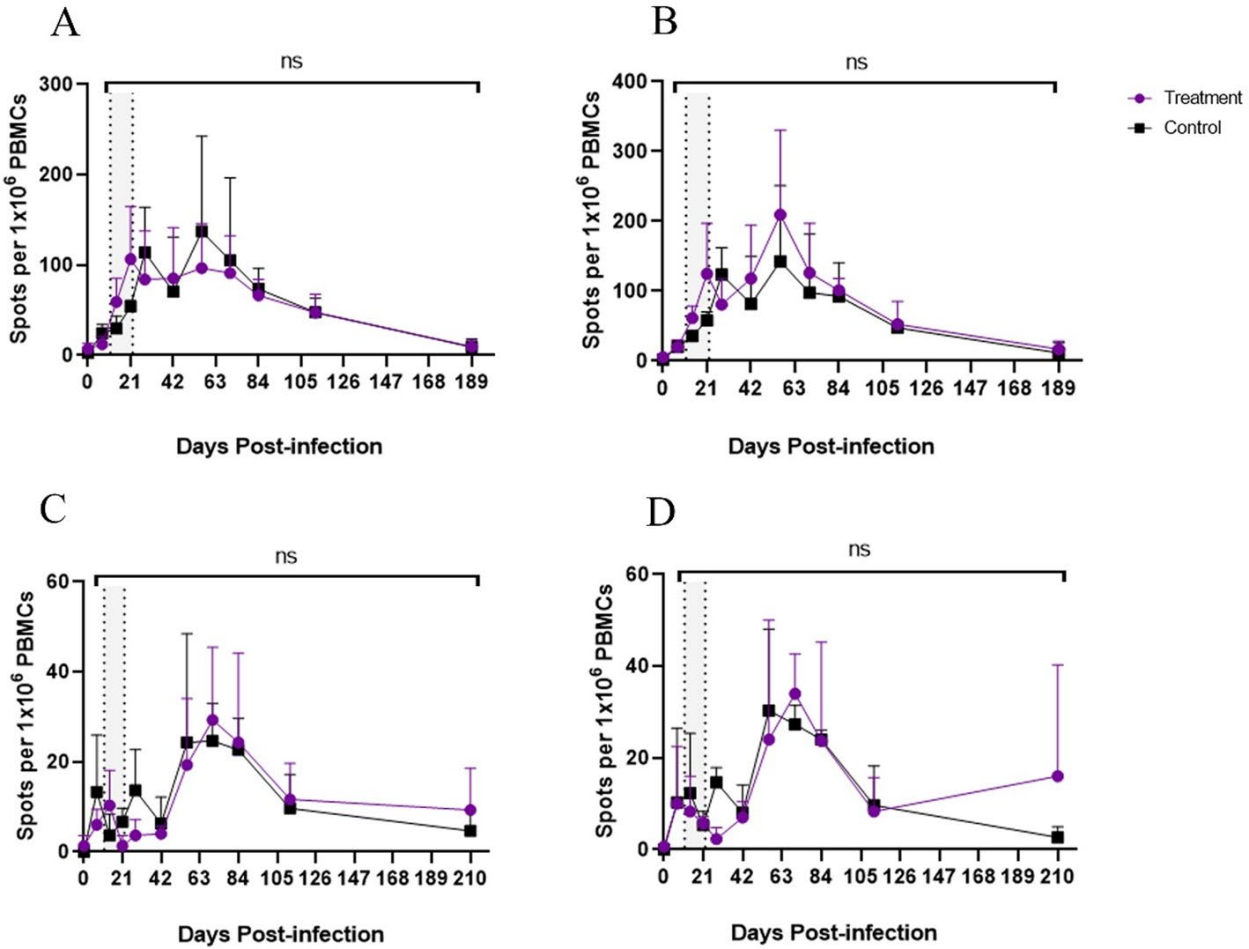
Cell-mediated immune response to MeV in macaques treated with remdesivir at onset of disease. Cells producing IFN-γ in response to: (**A**) pooled H glycoprotein peptides and (**B**) pooled N protein peptides. Cells producing IL-17 in response to: (**C**) pooled H glycoprotein peptides and (**D**) pooled N protein peptides as detected by ELISpot assay for late treatment group and control macaques. Shaded area indicates treatment period.

PBMCs were available for ELISpot T cell assays from the LT study group macaques and untreated controls and were used to quantify the cells producing IFN-γ and IL-17 after stimulation with H- or N- peptides (Fig. 7). Overall, no differences were noted in the numbers of MeV-specific cells producing IFN-γ or IL-17 during or after treatment.

## Discussion

Here we evaluated the in vivo activity of the small molecule antiviral remdesivir as a treatment for measles using a well-characterized rhesus macaque model. Treatment was administered intravenously for 12 days either as PEP beginning 3 days after infection, or as LT beginning at the time of disease onset 11 days after infection. The drug was well tolerated, and no evidence of toxicity was recognized. PEP treatment lowered levels of viral RNA in PBMCs during treatment, but RNA rebounded to levels similar to those of untreated macaques with cessation of treatment. Infectious virus was continuously recoverable when PBMCs of treated macaques were co-cultured with susceptible cells in vitro but the virus did not demonstrate reduced susceptibility to remdesivir. RNA was cleared more rapidly from lymphoid tissue, was variably detected in the respiratory tract and was not detectable in urine in PEP-treated macaques. Prophylactic treatment did not improve clinical disease scores or lymphopenia and reduced the quantity and quality of the antibody response to infection. Collectively, these data suggest that early treatment with remdesivir impacts the production of viral RNA consistent with the drug mechanism of action, but the 12-day course of PEP does not clear the infection as evident by the recovery of virus from PBMCs and rebound of viral RNA after treatment completion. In contrast, treatment at the time of disease onset (LT group) had little effect on levels of viral RNA or the antibody response and did not decrease clinical signs of disease. Therefore, based on the parameters studied, neither prophylaxis nor late treatment of measles with remdesivir improved outcomes in the rhesus macaque model of MeV infection.

Current recommendations for prophylaxis for unimmunized and vulnerable persons exposed to measles include immunization with a MeV-containing vaccine such as the measles, mumps, and rubella (MMR) vaccine within 72 h or intramuscular immunoglobulin (IMIG) within 6 days after exposure^25,26^. A study of unimmunized children in New York during a 2013 outbreak demonstrated that both interventions prevented measles with a measured effectiveness of 83.4% (confidence interval, CI 34.4, 95.8) for MMR vaccine and 100% (CI 56.2, 99.8) for IMIG^27^. PEP with remdesivir 3 days after infection did not prevent measles in susceptible rhesus macaques and therefore does not appear to be a potential substitute for currently approved approaches to prophylaxis.

Therapeutic options for acute measles are more limited than prophylaxis^28^ with the only recommended intervention being vitamin A, which lowers mortality independent of vitamin A deficiency by undefined mechanisms^29,30^. Established antivirals such as ribavirin are either not efficacious or are unreasonably toxic, and to date the polymerase and fusion inhibitor antivirals for MeV infection in development have not reached clinical trials^28^. Our studies of treatment of measles with remdesivir, an established broad-spectrum RNA-dependent RNA polymerase (RdRp) inhibitor, at the onset of clinical disease in immunologically competent rhesus macaques have proven to be safe, but with little effect on clinical disease or infectious virus clearance. Similar treatment studies of the experimental small molecule polymerase inhibitor ERDRP-0519 in MeV-infected squirrel monkeys (*Saimiri sciureus*) showed decreased MeV shedding from the respiratory tract without an effect on virus clearance from PBMCs or on clinical disease^31^ further suggesting the limited effects of treatment with direct acting antivirals at disease onset, a time when virus clearance has been initiated by the adaptive immune response. However, remdesivir treatment might facilitate virus clearance in immunocompromised individuals with persistent infection or from sites of virus persistence where immune-mediated viral clearance is less effective (*e.g.* brain for subacute sclerosing panencephalitis, SSPE), as suggested previously for Ebola virus persistence in semen^32^.

These studies of NHPs receiving prophylactic and therapeutic administration of polymerase inhibitors for measles identified potentially important consequences of in vivo therapy with direct acting antivirals. Despite suppression of viral RNA synthesis during treatment, macaques receiving remdesivir early after infection (DPI 3) continued to have detectable infectious virus on in vitro co-cultivation of PBMCs. Furthermore, after the 12 days of treatment ended, levels of viral RNA in PBMCs rebounded suggesting resumption of virus replication. The recovered virus remained susceptible to remdesivir. This indicates that antiviral treatment with remdesivir alone does not result in virus clearance or have a long-term effect on virus replication in PBMCs. It is not known whether longer treatment would be more effective. Previous studies of remdesivir treatment of Nipah virus-infected NHPs beginning 24 h after respiratory tract infection showed little treatment effect on levels of infectious virus or viral RNA in upper respiratory samples; however viremia was prevented and animals were followed for 3 months without evidence of relapse^12^. In NHPs infected intramuscularly with Ebola or Marburg virus, remdesivir decreased levels of viral RNA in plasma and improved survival with follow-up for 4–6 weeks^13,14^.

Remdesivir is the monophosphate prodrug for a 1’-cyano-substituted adenine nucleoside analog with broad activity against RNA viruses. It inhibits viral RNA synthesis by interacting with the RdRp nucleoside binding pocket, incorporation into viral RNA and inhibition of further RNA synthesis by delayed chain termination and by template-dependent inhibition of viral RNA replication^33–37^. Drug activity is cell-type dependent due to varied availability of enzymes for conversion to the active triphosphate form and remdesivir is metabolized to its active triphosphate nucleoside in macaque lymphocytes, a primary site for MeV replication^13,38^. Preclinical studies of ERDRP-0519, another small molecule RdRp inhibitor, for treatment of MeV-infected squirrel monkeys showed reduced levels of MeV RNA and infectious virus without evidence of relapse, ERDRP-0519 differs from remdesivir in that it binds to both the polymerase and capping domains of the MeV L polymerase protein to inhibit initiation of RNA synthesis as well as elongation and may have a more permanent effect on viral replication than remdesivir^31,39^. However, these studies were terminated 21 days after infection so effects on viral RNA clearance over a longer term were not evaluated. The durability of suppression of virus replication by direct acting antivirals and potential for viral rebound and recurrence of symptomatic disease after cessation of treatment^40^ merits further investigation.

The effect of remdesivir treatment on respiratory tract infection and MeV shedding noted a greater effect on levels of viral RNA in the lower respiratory tract (BAL fluids) than the upper respiratory tract (nasal swabs). This distinction was also noted in remdesivir efficacy studies in NHPs infected with MERS-CoV and SARS-CoV-2^15,16^ potentially suggesting a difference in the antiviral effects on infected epithelial cells in these locations or a differential distribution of the drug across the upper and lower respiratory tract.

Despite early initiation of the antiviral 3 days after infection and significantly decreased levels of viral RNA during treatment, there was no improvement in clinical disease or lymphopenia. Immune cells are a major target for WT MeV and effects of infection on the immune system can be profound. For instance, immune suppression resulting in an increased susceptibility to other infections is a major cause of mortality due to measles^41–44^. Early administration of remdesivir led to more rapid clearance of viral RNA from LNs, but not from BM, but the effect of treatment on immune suppression was not evaluated.

Prolonged persistence of viral RNA in lymphoid tissue has also been proposed to contribute to the durability of the immune response to MeV^2,45^. Viral RNA is detectable in lymphoid cells for months, despite clearance of the infectious virus^1,2^. In our studies, titers and maturation of antiviral antibody was impaired in PEP-treated macaques compared to both untreated macaques and those with late treatment. Titers of MeV-specific IgM, IgG and neutralizing antibodies were lower as was the avidity of MeV-specific IgG. Lower levels of antibody, particularly IgM is likely due to restricted expression of viral proteins for induction of the immune response and expansion of virus-specific lymphocytes and lower neutralizing antibody titers were also observed in NHPs treated with ERDRP-0519^31^. Impaired avidity maturation may reflect the more rapid clearance of viral RNA from lymphoid tissue resulting in less ongoing germinal center stimulation. Nevertheless, PEP-treated macaques produced neutralizing antibody titers predicted to protect from clinical measles^46,47^. Further examination of the effects of remdesivir treatment on immune suppression and long-term immunity to measles will be of interest.

Our study is limited by a small sample size due to the resources required to study macaques. Additionally, all assays could not be performed at all time points due to limitations in sample availability. Finally, although macaques are an excellent model for MeV infection, this study does not reflect the wide range of host diversity that could lead to differences in treatment effects in humans.

In summary, treatment of WT MeV-infected macaques with remdesivir is safe and decreases MeV replication but does not improve clinical outcome or prevent measles-induced lymphopenia. Infectious virus is still present in the infected cells and replication resumes once drug treatment is removed implying that infected macaques may still be infectious, even while being treated. Notably, MeV RNA, although suppressed during treatment, increases once treatment is stopped. The effect of this treatment on the durability of the immune response to MeV over time remains unknown, but despite lower titers of IgG and lower avidity maturation, levels of neutralizing antibodies predicted to be protective are still present 6 months after infection. Future studies evaluating how early suppression of MeV RNA affects the durability of the immune response and the susceptibility to other infections will be needed.

## Materials and methods

### Animals, infection, and sample collection

Fifteen (6 female, 9 male) 2- to 3-year-old Indian-origin rhesus macaques (*Macaca mulatta*) were obtained from Johns Hopkins University. All macaques were seronegative for measles, simian immunodeficiency virus, simian T-cell leukemia virus, and simian type D retrovirus prior to entering the study. The macaques were divided into PEP (N = 9) and LT (N = 6) study groups. The Bilthoven strain of WT MeV (genotype C2; gift of Albert Osterhaus, Erasmus University, Rotterdam, The Netherlands) was grown in phytohemagglutinin-stimulated human cord blood cells and assayed by plaque formation on Vero/hSLAM cells (gift from Yusuki Yanagi, Kyushu University, Fukuoka, Japan)^48^. Following baseline measurements, macaques were infected intratracheally with 10^4^ plaque-forming units (PFU) of MeV in 1 mL PBS. Macaques were singly housed in individually ventilated cages under ABSL2 + conditions (with relative negative air-pressure) for the duration of the infectious period (DPI 0 to 28) and then moved back out of isolation into routine cage housing in pairs for the duration of the study. On DPI 3, macaques in the PEP study group (N = 6; females 64E, 79F, 83F and males 94E, 35F, 41F) received an intravenous 10 mg/kg loading dose of remdesivir (Gilead Sciences Inc.), followed by daily 5 mg/kg maintenance doses on DPIs 4–14. Untreated control macaques (N = 3; female 80F and males 42F, 43F) received equal volumes of intravenous vehicle on the same days. For the treatment study group, remdesivir (N = 3; female 88F and males 57F, 90F) or vehicle (N = 3, female 92F and males 104F, 108F) infusions were as for the PEP study but initiated on DPI 11. The study is reported in accordance with ARRIVE guidelines.

Heparinized blood was collected from the femoral vein before infection and every 3–14 days after infection for up to 10 months to follow RNA persistence and immune parameters. Routine laboratory parameters during the acute phase of the study included complete blood counts with differentials and aminotransferase levels and were performed onsite using an IDEXX Procyte hematology analyzer and a DiaSys Respons 910 Vet analyzer. Clinical disease was scored using a protocol adapted from scoring parameters in previous remdesivir NHP treatment studies^12,13^ (Table S1). Remdesivir or vehicle dosing occurred after clinical scoring and after samples were collected. PBMCs and plasma were isolated by whole blood gradient centrifugation on Lympholyte-Mammal (Cedarlane Labs). Inguinal LN biopsies were performed, and BM was collected by aspiration from the femur or humerus into a heparinized syringe. BAL samples were obtained with a modified catheter mini-BAL method and nasopharyngeal samples were collected using nylon flocked swabs (Puritan Medical Products) and placed in viral transport medium. For all procedures, animals were anesthetized with intramuscular ketamine (5–10 mg/kg) or ketamine plus dexmedetomidine (0.025 mg/kg). All procedures were approved by the Johns Hopkins University Institutional Animal Care and Use Committee and conducted in accordance with guidelines in the Animal Welfare Regulations and the Guide for the Care and Use of Laboratory Animals within a fully AAALAC-accredited facility.

### Quantification of MeV N gene RNA

MeV RNA in PBMCs, LN, BM, BAL, and nasopharyngeal samples was quantified by qRT-PCR as previously described^1,22,49^. Briefly, RNA was isolated from 2 × 10^6^ PBMCs, BM cells or LN cells; 10^6^ BAL cells, and nasopharyngeal swab cell pellets. The MeV N gene was amplified (Applied Biosystems Prism 7700) using a one-step RT-PCR kit with TaqMan primers (MVN Forward: 5′-GGGTACCATCCTAGCCCAAATT-3′; MVN Reverse: 5′-CGAATCAGCTGCCGTGTCT-3′) and probe (5′-CTCGCAAAGGCGGTTACGGCC). Controls included GAPDH amplification (Applied Biosystems) and no template controls. Copy number was determined by construction of a standard curve from 10^0^ to 10^8^ copies of RNA synthesized by in vitro transcription from a plasmid encoding the Edmonston MeV N gene (MV41 5′-CATTACATCAGGATCCGG-3′; MV42 5′-GTATTGGTCCGCCTCATC-3′). Data were normalized to the GAPDH control and expressed as [(copies of MeV N RNA)/(copies of GAPDH RNA)] × 5E10.

### Infectious virus assays

PBMC and LN cells (0.5 × 10^6^) were tenfold serially diluted and co-cultivated with 50% confluent Vero/hSLAM cells in quadruplicate in the absence of remdesivir for 4–5 days and scored for syncytia formation. Results are reported as the log_10_(TCID_50_). To recover virus for drug sensitivity testing, supernatant fluids from cocultures were collected after 4 days and used to infect Vero/hSLAM cells. After infection with virus isolated from the PBMCs of one of two macaques (PEP-treated 83F and untreated control 43F) for 1 h, cells were washed and treated with remdesivir (1 µM) or vehicle control. After 4 days, virus produced was quantified by syncytia formation on Vero/hSLAM cells to determine TCID_50_ and percent inhibition was calculated.

### Antibody assays

Enzyme immunoassays (EIAs) were used to measure MeV-specific immunoglobulin (Ig)M and IgG in plasma. Maxisorp 96-well plates (Nalgene Nunc International) coated overnight with lysate from MeV-infected Vero cells (5 μg/mL, Advanced Biotechnologies), lysate from MeV H-expressing L cells (1:1000)^50^ or baculovirus-expressed MeV N (1:2000)^51^ were blocked with 5% nonfat dry milk in 0.05% Tween-20 in PBS for 3 h at room temperature for IgM and 37 °C for IgG. Plasma was serially diluted twofold for IgG (1:50 to 1:102,400) or 1:100 for IgM, added to the coated plate and incubated at room temperature for 2 h. The secondary antibody was horseradish peroxidase-conjugated–goat anti–monkey IgM or IgG (MilliporeSigma). Plates were developed using 3,3′,5,5′-tetramethylbenzidine as the substrate and the reaction was stopped using 2 M H_2_SO_4_. Plates were read at 450 nm. For IgG the EIA titer was determined as the highest dilution with an optical density (OD) three times background. The IgM results are reported at OD 450 nm.

To assess the avidity of MeV-specific IgG antibody, EIAs were modified by addition of increasing concentrations (0.5 to 4 M) of ammonium thiocyanate (NH_4_SCN) for 15 min after plasma incubation to disrupt the antigen–antibody interaction. The avidity index was calculated as the concentration of NH_4_SCN required to remove 50% of bound antibody (AI_50_).

Neutralizing antibody was measured by a plaque reduction assay. The Edmonston strain of MeV was mixed with serially diluted plasma (1:8 to 1:262,144) and assayed for plaque formation on Vero cells (ATCC). Data are reported as the reciprocal of the serum dilution at which the number of plaques is reduced by 50% (PRNT_50_).

### Enzyme-linked immunosorbent spot (ELISpot) assays

ELISpot assays were used to measure MeV-specific IFN-γ and IL-17 producing cells. Multiscreen HTS HA Opaque 96-well filtration plates (Millipore) were coated with antibody to human/monkey IFN-γ (MabTech, 3421 M-3–1000; 5 µg/ml) or IL-17A (MabTech, mAb MT241; 10 µg/ml) at 4 °C overnight. PBMCs were thawed, washed, resuspended in culture medium, and rested overnight at 37 °C/ 5% CO_2_. Plates were washed with 1X PBS and then blocked with RPMI/10% FBS for 2 h at 37 °C/5% CO_2_. PBMCs were counted and transferred to ELISPOT plates. PBMCs were not stimulated or stimulated with pooled MeV H or N peptides (5 µg/ml) or concanavalin A (ConA, 5 µg/ml). 10^5^ PBMCs were added to wells stimulated with ConA; 1–2.5 × 10^5^ PBMCs were added to unstimulated, H and N peptide-stimulated IFN-γ wells; 5 × 10^5^ PBMCs were added to unstimulated, H and N peptide-stimulated IL-17A wells. Plates were incubated at 37 °C/5%CO_2_ for 40–42 h. Biotinylated anti-human IFN-γ (Mabtech, mAb 7-B6-1; 1 µg/ml) or anti-human IL-17A (Mabtech, mAb MT504; 1 µg/ml) antibody was added for 2 h. Plates were developed with streptavidin-HRP (MabTech; 1:1000) for 1 h and diaminobenzidine substrate (Vector Laboratories) for 20 min. Plates were read and analyzed using an ImmunoSpot plate reader and BioSpot 5.0.6 software (C.T.L.). Data are presented as total spot-forming cells (SFCs)/10^6^ PBMCs. All assays were done in duplicate.

### Statistics

All statistical analyses were performed with GraphPad Prism version 9.4.1. A 2-way ANOVA was used for all statistical analyses between groups and a Mann–Whitney test was used for comparisons of samples on a specific day. *p* values less than 0.05 were considered significant. Data are presented as the mean values ± SD.

### Study approval

All studies were performed in accordance with experimental protocols approved by the Johns Hopkins University Animal Care and Use Committee (Protocol PR19H316).

## Supplementary Information

Supplementary Information.

## Data Availability

The datasets analyzed during the current study are available from the corresponding author on reasonable request.
