# Supplementary material for: Effect of remdesivir post-exposure prophylaxis and treatment on pathogenesis of measles in rhesus macaques
Source: Sci Rep. 2023 Apr 20;13:6463. doi: 10.1038/s41598-023-33572-7 (PMC10116456; doi:10.1038/s41598-023-33572-7)
Supplement: Supplementary file 1 — Supplementary Information. [file 41598_2023_33572_MOESM1_ESM.docx]

**SUPPLEMENTARY MATERIAL**

**Table S1.** Clinical score parameters.

| Parameter | Degree | Points |
| --- | --- | --- |
| Energy | Bright, alert, responsive | 0 |
|  | Quiet, alert, responsive | 1 |
| Mentation | Normal (Tearing papers, approaches for treat) | 0 |
|  | Depressed (Decreased engagement) | 1 |
| Appetite | Normal (>90%) | 0 |
|  | Decreased (50-90% meal) | 1 |
|  | Very decreased (<50% meal) | 2 |
| Rash | None | 0 |
|  | Mild (<50% body) | 1 |
|  | Severe (>50% body) | 2 |
| Fever | <103F | 0 |
|  | >103F | 1 |
|  | Documented temp |  |
| Conjunctivitis | None | 0 |
|  | Present | 1 |
| Koplik spots | None | 0 |
|  | Present | 1 |
| Lymphadenopathy | None | 0 |
|  | <1 cm | 1 |
|  | >1 cm | 2 |
| Urine output | Normal | 0 |
|  | Decreased | 1 |
| Stool | Normal | 0 |
|  | Diarrhea | 1 |
| Other/Notes |  |  |


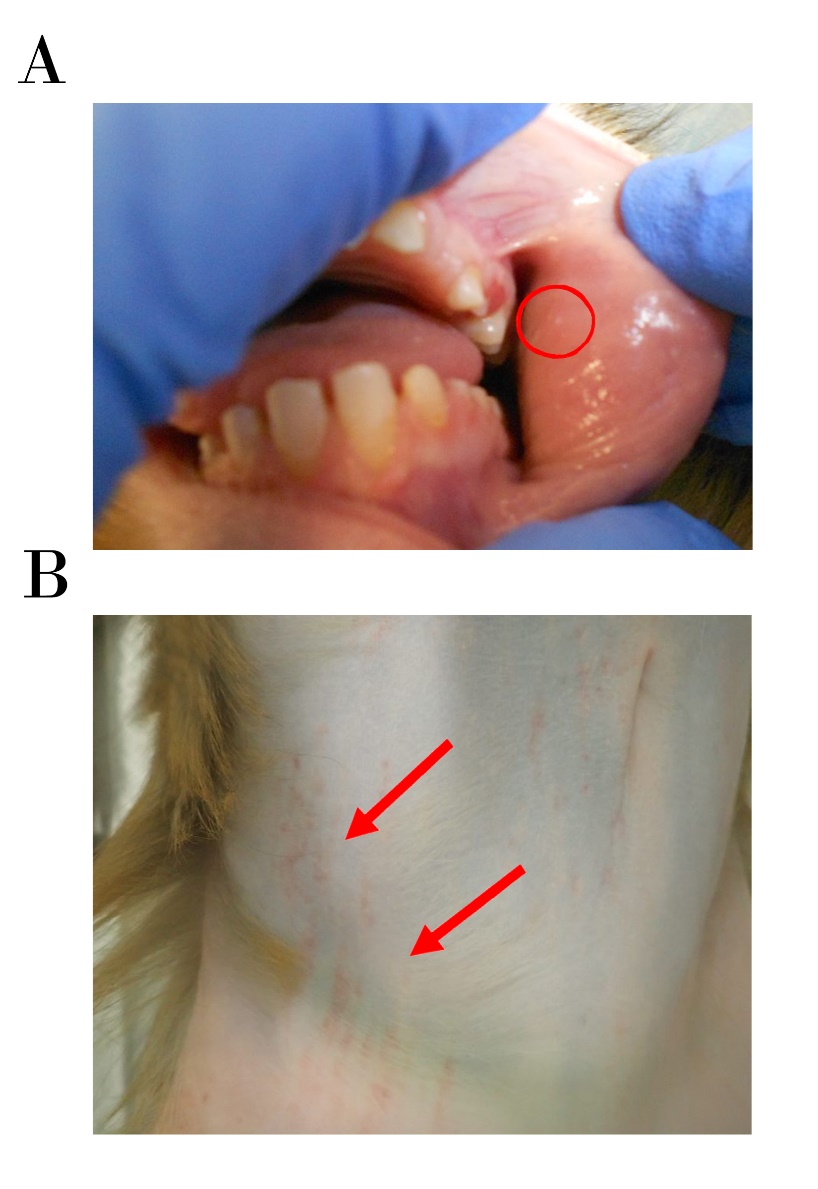


**Fig. S1.** Images of clinical findings seen on macaques infected with wild-type MeV. **(A)** Koplik spots (red circles) and **(B)** rash (red arrows).

**
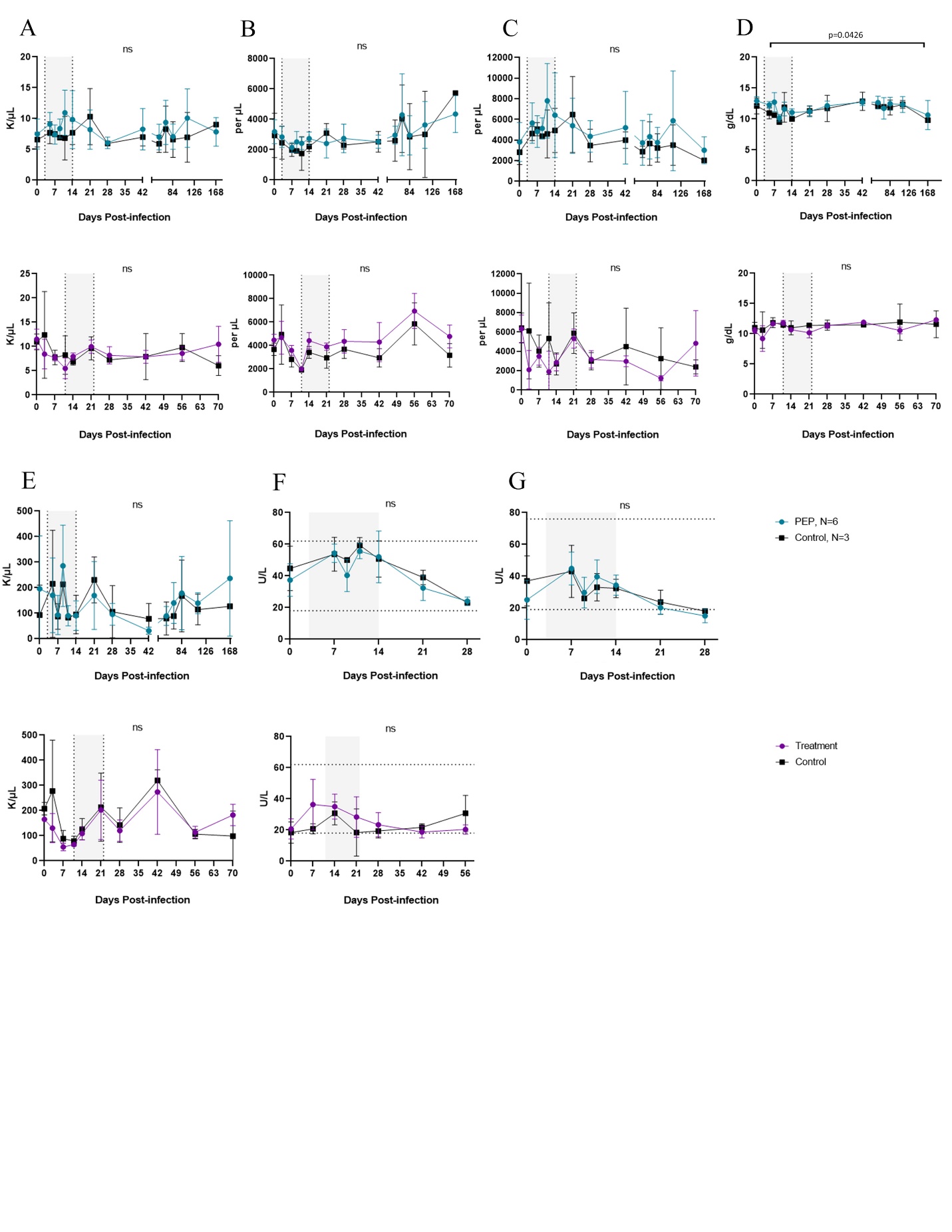
**

**Fig. S2.** Laboratory findings. Blood was drawn for complete blood counts and aminotransferase levels at varying days post infection (DPI) PEP study group (top rows) and LT study group (bottom rows) macaques. Shaded areas indicate treatment period. Horizontal lines indicate approximate normal ranges of aminotransferases for macaques. **(A)** White blood cell count, **(B)** Absolute lymphocyte count, **(C)** Absolute neutrophil count, **(D)** Hemoglobin, **(E)** Platelets, **(F)** Aspartate aminotransferase, **(G)** Alanine aminotransferase.


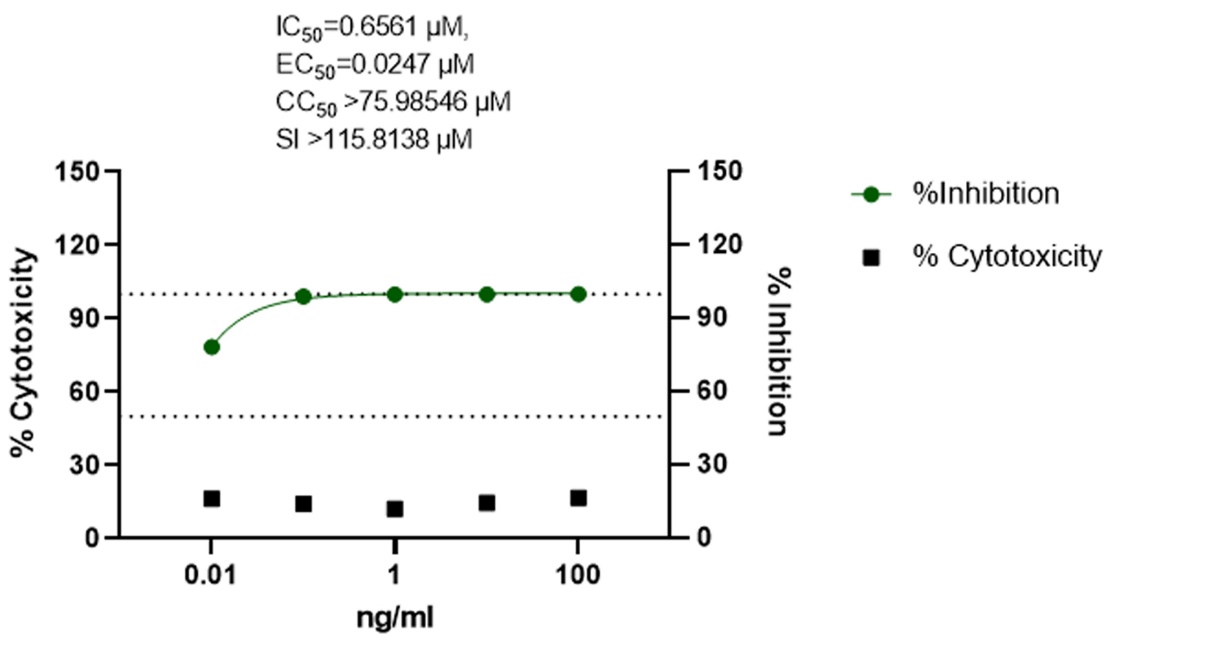


**Fig. S3.** Inhibition and cytotoxicity of remdesivir in vitro against a wild type strain of MeV (Bilthoven strain). IC_50_=Inhibitory concentration, 50%; EC_50_=Effective concentration, 50%; CC_50_=cytotoxic concentration, 50%; SI=selectivity index.
